# Miniaturized Real-Time PCR on a Q3 System for Rapid *KRAS* Genotyping

**DOI:** 10.3390/s17040831

**Published:** 2017-04-11

**Authors:** Maria Guarnaccia, Rosario Iemmolo, Salvatore Petralia, Sabrina Conoci, Sebastiano Cavallaro

**Affiliations:** 1 Institute of Neurological Sciences, Italian National Research Council, Via Paolo Gaifami, 18-95125 Catania, Italy; maria.guarnaccia@cnr.it (M.G.); iemmolo.rosario@gmail.com (R.I.); 2 STMicroelectronics, Stradale Primosole, 50-95121 Catania, Italy; sabrina.conoci@st.com

**Keywords:** qPCR, Genome-based test, diagnostics, colorectal cancer

## Abstract

Colorectal cancer (CRC) is an aggressive human malignancy with a complex genomic landscape harboring *KRAS* mutations. In 40%–60% of patients with CRC, constantly active *KRAS* proteins affect the prognosis, surgical strategy, and clinical benefit from therapy with anti-epidermal growth factor receptor (EGFR) agents. For this reason, there is a greater demand for minimally-invasive diagnostic devices to characterize the genetic pattern and prevent the acquired mechanism to drug resistance. The rapid developments in cutting-edge diagnostic techniques are expected to play a growing role in medicine and represent an attractive promise to identify potential responders to personalized medicine. Here we propose a new method to simultaneously detect the main *KRAS* mutations on the portable real-time PCR Q3 platform. This platform is based on hybrid silicon-plastic technology implemented in a miniaturized chip able to achieve a sample-in answer-out rapid analysis, allowing a new approach to genetic counseling and testing.

## 1. Introduction

Colorectal cancer (CRC) is one of the main worldwide health problems owing to its high prevalence and mortality rates [1]. The pathogenesis of CRC is a complex process characterized by genetic and epigenetic alterations, transforming normal into cancer cells. Several genes associated with CRC risk have been identified, but members of the Ras superfamily, which includes the Kirsten rat sarcoma viral oncogene homolog (*KRAS*), Harvey rat sarcoma viral oncogene homolog (HRAS), and neuroblastoma RAS (NRAS) oncogenes, still constitute the most frequently mutated oncogenes, with a prevalence of 50% [2,3,4]. With the prevalence rates of 49.5%, the mutations in the *KRAS* gene are the most common among the RAS mutations in cancer [3,5]. Located on chromosome 12p12, *KRAS* is involved in the G-protein signal transduction pathway modulating cellular proliferation and differentiation [6]. The most frequent mutations in *KRAS* are single base missense mutations, 98% of which are found at residues G12 and G13 of codons 12 and 13, respectively [7,8]. The constitutive activation of *KRAS*, due to these mutations, impairs the ability of GTPase activating proteins to hydrolyze *KRAS*-bound GTP [4]. This leads to the activation of multiple downstream proliferative signaling pathways, such as mitogen-activated protein kinase (MAPK), the RAF/MEK/ERK signaling cascade, the PI3K/AKT signaling cascade, and the RAL/GDS pathway [9]. In addition to the most frequent hotspot mutations, other specific *KRAS* mutations are associated with a more aggressive tumor phenotype and with risk to develop resistance to therapy [10]. Mutations in codon 61 (exon 3) and codon 146 (exon 4), for example, result in an increased levels of guanosine triphosphate-bound RAS proteins and are associated with acquired resistance to EGFR antibodies and clinical outcome in CRC patients [11,12].

The guidelines for CRC screening recommend multiple options that differ with regard to their effectiveness, risk, and degree of invasiveness. Conventionally, people with symptoms or signs that suggest the presence of colorectal cancer or polyps are investigated by abdominal computed tomography or invasive screening tests, such as double-contrast barium enema (DCBE), flexible sigmoidoscopy (FS), or colonoscopy [13,14]. The latter technique is the most effective standard method and prevention tool in CRC screening, but the high cost and adverse events limits the diagnostic value [15,16]. Serum levels of carcinoembryonic antigen or cancer antigen 19-9 are used as biomarkers for CRC to monitor the progression, metastasis development, and therapy efficacy. However, the evaluation of tumor marker concentrations has poor sensitivity and specificity for screening at-risk populations and can produce false-positive test results [17,18]. The molecular approaches to CRC screening, including high-resolution melting analysis, pyrosequencing, or Sanger sequencing, have the advantages of high sensitivity for mutant detection, but require skilled personnel, high costs, and specialized equipment [8,19]. Furthermore, the problem of patient management regarding the selection of the most proper strategy to chemotherapy has to be solved. Several studies investigating the positive response or side effects of therapeutic agents, such as Irinotecan and Oxaliplatin, and epidermal growth-factor receptor (EGFR) monoclonal antibodies (e.g., Cetuximab, Panitumumab), have highlighted the relationship between immunotherapy resistance and the expression of several oncogenes, such as *KRAS* [20,21]. 

The ideal screening programs should allow to classify the individual patient’s risk level on the basis of personal, family, and medical history, and to identify who require further investigation in order to recommend preventive strategies or treatment. Moreover, the test must be reliable enough, precise and cost-effective [22]. In this regard, newly-developed biosensor technologies, based on genomic approaches, offer new options in the field of genetic screening [23]. The major features of these micro- and nanofabrication technologies are the low-cost mass production, user-friendly miniaturization, speed of analysis, and greater accuracy in terms of single molecule detection [24,25].

In this study, we present an optimized assay for qualitative, quantitative, and quick screening of *KRAS* in clinical samples. The assay was implemented on the Q3 System*,* developed by STMicroelectronics for ease of use by non-specialist personnel as a point-of-care instrument. The Q3 platform is a system based on silicon microchip, integrating temperature sensors and heaters able to address the quantitative and qualitative identification of specific nucleic acid sequences through real-time polymerase chain reaction (PCR) amplification [26]. The platform is completed by a customized instrument that thermally and optically drives the chip during the real-time PCR process and a dedicated software package for data analysis [27].

Taking advantage of the STMicroelectronics RT-PCR technology, we aimed to develop a new real-time-based assay able to identify the eight mutations of *KRAS* occurring in codons 12 and 13 of exon 2.

## 2. Materials and Methods 

### 2.1. Primers and Probes Design

The assay for detecting the panel of *KRAS* (accession number NG_007524) mutations includes the eight most common, spanning in codons 12 and 13 of exon 2 (c.34G>A; c.34G>C; c.34G>T; c.35G>A; c.35G>C; c.35G>T; c.38G>A, c.33_34insGGAGCT). High-quality primers for each mutation were designed using free design tools (Primer-Blast, NCBI, Bethesda, MD, USA). Primers were selected with an optimal length of 24–28 bp, a content of 50% of CG and an optimal real-time PCR annealing temperature between 59 °C and 61 °C. The specificity to the target sequence was verified in silico with the UCSC Genome Browser and Primer-BLAST. The allele-specific 3’ terminus of each primers was adapted to anneal specifically the mutated DNA template. To promote high-efficiency of amplification, amplicons in the 70–100 bp range were selected.

The oligonucleotide probes common for the wild-type and mutant *KRAS* alleles were designed. The probes that hybridize to an internal sequence were labeled at the 5’ end with the FAM (6-carboxyfluorescein) dye.

To achieve the best device performance, different probes, primer concentrations and melting temperatures were evaluated. The amplification test was performed using a conventional real-time PCR system (LightCycler 1.5, Roche Diagnostics, Indianapolis, IN, USA). Primer and probe sequences are listed in Table 1. 

### 2.2. Real-Time PCR Q3 Platform

The real-time PCR Q3 Platform includes three main components: (a) a disposable silicon-based microchip; (b) a proprietary instrument (Q3 instrument); and (c) a customized software package (*Q3* software). Below are the main technical features of the Q3 components.

**Figure 1:**
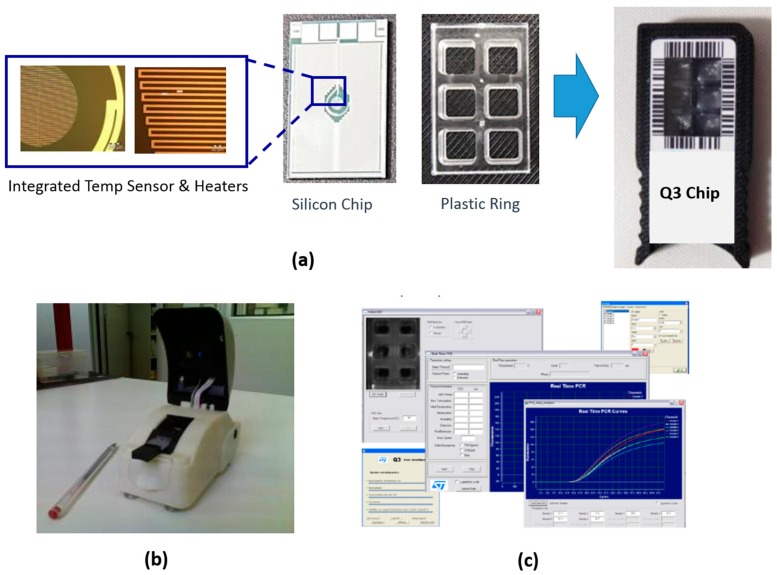
Real-time PCR Q3 platform: (**a**): disposable silicon-based microchip; (**b**) Q3 instrument; and (**c**) customized software package (Q3 software).

Disposable silicon-based microchip*.* This is manufactured by silicon-plastic hybrid technology. The bottom part is a silicon chip (17 mm × 13 mm) with on-board integrated temperature sensors and heaters. The top part is a polycarbonate ring defining six reaction chambers of 27 µL each. Top and bottom parts are glued by Delo adhesive (DELO Industrial Adhesives) and assembled in a plastic holder (Figure 1a).

The Q3 instrument is a compact tool (14 cm × 7.1 cm × 8.7 cm) (Figure 1b) that thermally and optically drives the microchip during the real-time PCR process. The optical module includes two independent optical channels, for FAM and VIC fluorescent reporters. Their excitation is done through LED light sources centered at a wavelength of 470 nm and 530 nm, respectively, while the emission is monitored by a CCD camera with a high-pass filter at 520 nm or 20 nm wide band-pass filter centered at 556 nm. The thermal module is integrated to drive the temperature sensors and heaters of the microchip during real-time PCR reaching the following temperature performance characteristics: (a) temperature control accuracy: ±0.2 °C; (b) heating rate: 15 °C/s; (c) cooling rate: 8 °C/s; and (d) temperature resolution: 0.1 °C.

The Q3 software package manages both the optical and thermal modules for the real-time PCR process allowing easy programming and carrying out data capturing and analysis at the end of the real-time PCR run giving the final amplification curves and Ct values (Figure 1c).

### 2.3. KRAS Genotyping on Real-Time PCR Q3 Platform

DNA obtained from tumor samples was used as a template in all real-time PCR assays. In order to standardize the DNA extraction and facilitate the collection of high-quality material, we used an automated workstation and standard protocols (EZ1, Qiagen, Germany). 

The quality control of extracted DNA was performed by spectrophotometer analysis. The mutational status of *KRAS* in collected samples was analyzed by direct sequencing. 

A master mix of real-time PCR assays was prepared to contain 10 ng of reference DNA, 10 µM of reverse and allele-specific forward primers, 100 nmol/L of FAM-labeled *KRAS* probe, 5 μL of 1X TaqMan Genotyping Master Mix, and H_2_O to a final volume of 6 µL per chamber. The wells of the silicon chip were covered with a dry film to prevent reagent evaporation. Thermocycling conditions were the following: an initial denaturation step at 97° × 10′ (one cycle), followed by 45 cycles at 97° × 15″ and 64° × 60″. In every run, positive and negative control samples were co-amplified and the Ct values obtained from the portable real-time PCR were compared. Region of Interest (ROI) areas in the microchip were manually selected by the operator to acquire, at the end of the experiment, the fluorescent signal of each single well in which the real-time PCR reaction occurs. The positions of the ROIs are highlighted on the image by a green circle whose size and position can be adjusted. The Q3 software allows a correction to the ROI parameters at the end of the analysis.

### 2.4. Assay Validation

To determine the performance and efficiency of the Q3 system, the PCR protocol was implemented in a commercial real-time PCR system (LightCycler 1.5, Roche Diagnostics, USA). The following amplification program was used: an initial denaturation step at 97° × 10′ (one cycle), followed by 45 cycles of 95° × 15″, 62° × 10″, and 72° for 10 min, with a final extension step at 72 °C for 5 min. In addition, the limit of detection was assessed according to standard guidelines. The efficiency and sensibility were calculated using both 10-fold and two-fold dilution series of DNA templates. 

## 3. Results

To identify the most common point mutations of *KRAS*, a specific real-time PCR assay was developed using the Q3 platform. Primers and probes were designed and evaluated by conventional PCR before performing the experimental protocol on the disposable cartridge of the Q3 system. At the end of the real-time PCR, the dedicated Q3 software produces a graphical output where DNA detection is expressed by cycle threshold (Ct) values. This software was designed to enable fast and highly accurate data analysis of the amplified product. As in a standard method, the algorithm for Ct values calculates the cycle at which the individual PCR amplification reaches a significant threshold. As the calculated Ct value is proportional to the number of target copies present in the sample, the Ct value is a precise quantitative measurement of the presence of the respective target that the primer sets were designed to recognize.

**Figure 2:**
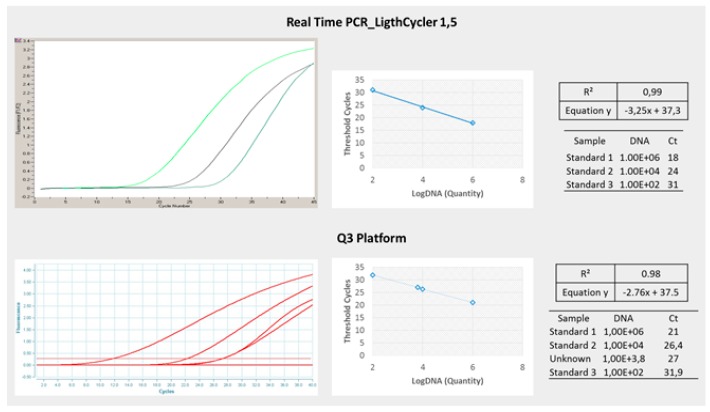
Real-time quantitative amplification results with a LightCycler 1.5 instrument and the Q3 system; dilutions of known concentrations of wild-type DNA were tested.

**Figure 3:**
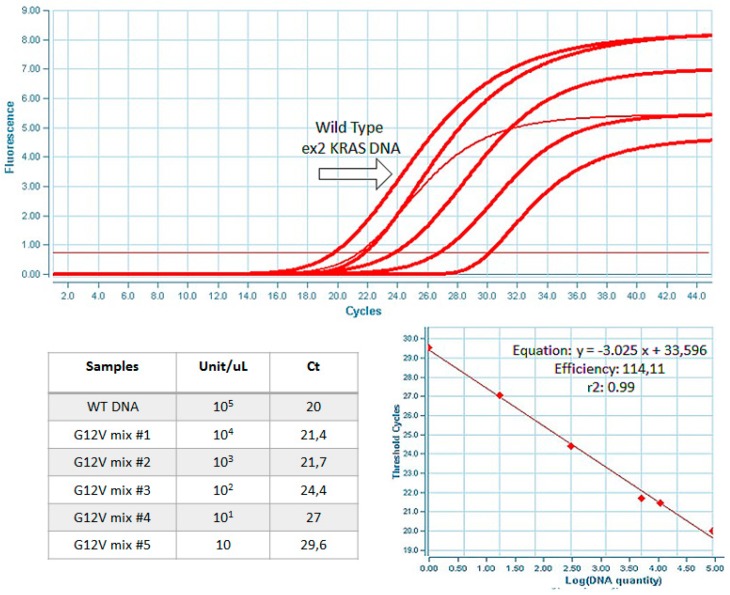
Analysis of PCR sensitivity using serial dilutions of *Kras*-mutated DNA in a background wild-type.

The sensitivity, selectivity, and specificity of the Q3 platform were compared to those obtained in a conventional instrument with the same protocols. As shown in Figure 2, the results obtained in the LightCycler 1.5 real-time PCR system and Q3 system were comparable in terms of threshold cycles (Cts) and amplification plots. The DNA amplification efficiency was estimated calculating the slope of the standard curve and the R2 value by using the method previously described [27]. The ROI area was aligned in order to correct the fluorescence value and avoid reading errors. In the Q3 system, the coefficient of correlation was estimated to be >0.98 with a slope of −2.76, while in the LightCycler 1.5 the R2 was >0.99 with a slope of −3.25. With regard to the limit of detection, the Q3 assay was able to detect up to one molecule of mutant DNA over a background of 100,000 wild-type molecules (Figure 3).

**Figure 4:**
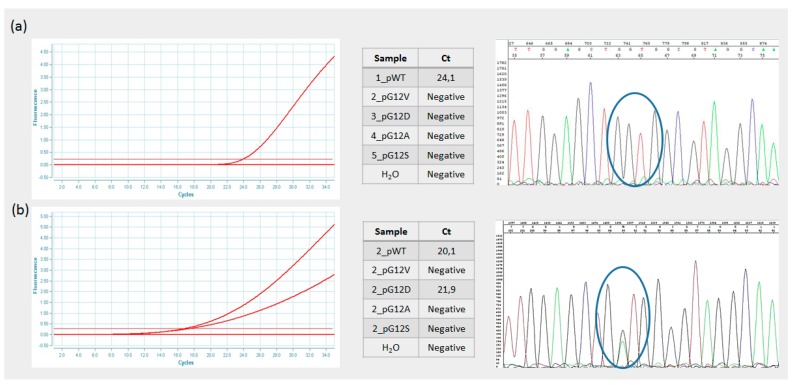
Validation of real-time Q3 assay using Sanger sequencing. Panel (**a**) shows the detection of a wild-type DNA; Panel (**b**) shows the detection of a mutant DNA sequence.

The genetic profile of each sample was evaluated by automated DNA sequencing. As shown in Figure 4, these results confirmed those obtained by Q3 real-time PCR analysis. Taken together, the obtained results indicate that the efficiency of the developed real-time chip was comparable to that of a conventional real-time PCR instrument and that the assay developed provided the correct mutational profile of the clinical samples.

## 4. Discussion

*KRAS* is one of the most common oncogenes involved in the multi-step process of cancer development, including cancer initiation, metastasis, and prognosis. Since *KRAS* mutations have emerged as frequent drivers of acquired resistance to anti-EGFR antibodies in CRC [28,29], their identification is crucial for diagnosis, monitoring, and treatment plan definition. In this manuscript we described a rapid and efficient method to perform a micro real-time PCR assay on the *Q3 system* for *KRAS* genotyping. The developed assay identifies the mutations in the *KRAS* that have a high value both for the clinical and supportive aspects of treatment [7]. The goal was to provide a quicker and cheaper *KRAS* mutation test, allowing processing and analysis of small samples. The assay allows for obtaining a molecular profile of patients and support treatment decisions. 

In this paper, we demonstrated the advantages of the Q3 platform in terms of diagnostic speed and sensitivity. The results achieved can be translated into opportunities in several fields, such as clinical practice, genetic screening, or epidemiology [30]. The assay developed with the Q3 system allows the molecular classification of CRC with greater advantages for the molecular pathological epidemiology (MPE) and for the application of personalized treatment strategies [30,31].

## 5. Conclusions

The ability to perform genetic screening on a small scale using a miniaturized device is an attractive promise to replace traditional experimental approaches. In recent years, the considerable progress of micro-technologies is being translated to medicine and supporting the development of personalized treatment approaches [32]. The clinical utility of an ultra-fast device is particularly evident for detection of actionable mutations, such as those occurring in the *KRAS* oncogene. The future challenge will be the translation of point-of-care devices, such as the one described in this study, into clinical practice for rapid and accurate disease management [33].

## Figures and Tables

**Table 1:** Primers and probe used for detecting the most common mutations in codons 12 and 13 of the *KRAS* gene on the Q3 system.

Primer	Sequence	Product Length (bp)
***KRAS-F***	*5’-GACTGAATATAAACTTGTGGTAGTTGGA-3’*	*98*
***KRAS-R***	*5’-CATATTCGTCCACAAAATGATTCTGA-3’*	
***G12S***	*5’-AATATAAACTTGTGGTAGTTGGAGCTA-3’*	*90*
***G12R***	*5’-AATATAAACTTGTGGTAGTTGGAGCTC-3’*	*90*
***G12C***	*5’-AATATAAACTTGTGGTAGTTGGAGCTT-3’*	*90*
***G12D***	*5’-AACTTGTGGTAGTTGGAGCTGA-3’*	*84*
***G12A***	*5’-CTTGTGGTAGTTGGAGCTGC-3’*	*82*
***G12V***	*5’-ACTTGTGGTAGTTGGAGCTGT-3’*	*83*
***G13D***	*5’-GTGGTAGTTGGAGCTGGTGA-3’*	*79*
**Probe**	Sequence	
***KRAS***	*5’-FAM-CTGTATCGTCAAGGCACTCT-MGB-3’*	
